# Novel Insights on Nitric Oxide Synthase and NO Signaling in Ascidian Metamorphosis

**DOI:** 10.3390/ijms23073505

**Published:** 2022-03-23

**Authors:** Annamaria Locascio, Quirino Attilio Vassalli, Immacolata Castellano, Anna Palumbo

**Affiliations:** 1 Department of Biology and Evolution of Marine Organisms, Stazione Zoologica Anton Dohrn, Villa Comunale, 80121 Napoli, Italy; quirino.attilio@gmail.com; 2 Department of Molecular Medicine and Medical Biotechnology, University of Naples Federico II, Via Pansini 5, 80131 Napoli, Italy; immacolata.castellano@unina.it

**Keywords:** *Ciona robusta*, nitric oxide synthase, NO signaling, tail regression, larval development, swimming

## Abstract

Nitric oxide (NO) is a pivotal signaling molecule involved in a wide range of physiological and pathological processes. We investigated NOS/NO localization patterns during the different stages of larval development in the ascidia *Ciona robusta* and evidenced a specific and temporally controlled pattern. NOS/NO expression starts in the most anterior sensory structures of the early larva and progressively moves towards the caudal portion as larval development and metamorphosis proceeds. We here highlight the pattern of NOS/NO expression in the central and peripheral nervous system of *Ciona* larvae which precisely follows the progression of neural signals of the central pattern generator necessary for the control of the movements of the larva towards the substrate. This highly dynamic localization profile perfectly matches with the central role played by NO from the first phase of settlement induction to the next control of swimming behavior, adhesion to substrate and progressive tissue resorption and reorganization of metamorphosis itself.

## 1. Introduction

Nitric oxide (NO) is a peculiar signaling molecule first discovered in mammals as a potent vasodilator, and extensively investigated in different species throughout metazoan evolution [1,2]. Nitric oxide synthase (NOS) is the enzyme responsible for its biosynthesis. In most invertebrates only one *Nos* gene is present, whereas in mammals three *Nos* gene copies have been identified, encoding for NOS1 (neuronal), NOS2 (inducible), and NOS3 (endothelial). These enzymes exhibit specific cellular/subcellular localization, regulation, and catalytic properties [3,4]. Analysis of gene organization and protein domains have revealed the extreme conservation of NOS structure in metazoans, from placozoans to mammals [1]. In addition, phylogenetic and syntenic analyses have provided novel insights into the origin of NOS proteins, suggesting that evolution was the result of multiple gene and genome duplication events together with changes in protein architecture [1]. Whereas in mammals NO acts mainly as neurotransmitter, vasodilator, and immune response mediator, in marine invertebrates the gas is involved in a variety of biological processes, including stress response to environmental pollutants or toxins [5,6,7,8,9,10], defense [11,12,13,14,15,16,17,18,19,20,21,22,23], neurotransmission [13,24], swimming [25], feeding [26,27], symbiosis [28], and fertilization and development [29,30,31,32].

During development, NO is involved in metamorphosis, a biological process by which the animal’s body structure changes rapidly with possible consequences on nutrition and behavior. Pharmacological manipulation experiments on NO signaling have evidenced that the regulatory role of NO on larval settlement and metamorphosis is highly conserved throughout evolution from sponges to chordates [33,34,35,36,37,38,39,40,41,42,43,44,45,46,47,48]. As reported in other species, in ascidians NO has been shown to act both as a negative regulator, repressing metamorphosis (*Boltenia villosa* and *Cnemidocarpa. finmarkiensis*) [34] or as a positive regulator, inducing the same process (*Herdmania momus* and *Ciona robusta*) [43,45]. Therefore, the picture emerging from the literature is very complex and probably a fine regulation of NO levels could differently affect the same biological processes during larval metamorphosis. In this respect, measurements of endogenous NO levels after treatments with NOS inhibitors or NO donors should be checked to rationalize apparently contrasting results [45].

An aspect deserving particular attention is NOS regulation and in particular regulation of NOS1, the predominant enzyme in invertebrates. The regulation of NOS1 is extremely complex and has been extensively investigated in mammals. The human *Nos* gene produces multiple mRNA isoforms with tissue-specific expression [49,50,51]. By contrast, few studies have been performed on NOS regulation in invertebrates, mainly in terrestrial and freshwater species. Interestingly, in the insect *Anopheles stephensi* the complexity of *Nos* transcriptional regulation resembles, to some extent, the situation in mammals [52]. In the mollusk *Lymnaea stagnalis*, NOS was regulated during long-term memory formation by an antisense mechanism involving *Nos* pseudogenes derived from DNA inversion and leading to nonfunctional NOS proteins [53,54]. The existence of enzymatically inactive NOS forms capable of acting as natural dominant negative regulators of NOS activity has also been reported during *Drosophila melanogaster* development [55].

Recently, common mechanisms of NOS regulation in non-vertebrate chordates were disclosed [32]. Putative regulatory regions of *Nos* identified in *Branchiostoma lanceolatum* and *C. robusta* were shown to function as enhancers during *Ciona* development. From a phylogenetic point of view, *C. robusta* is a hermaphroditic broadcast chordate spawner located just before vertebrate divergence. This is a strategic position from which to perform the evolutionary comparison of specific signaling pathways and to try to understand the mechanisms that led to the acquisition of novel vertebrate functions. *Ciona* has a biphasic life cycle with a simple swimming tadpole larva and a sessile juvenile/adult. The complexity of the processes controlling its development was extensively investigated, and a series of cellular and molecular events were identified [56,57,58,59]. *Ciona* represents a useful model system to better understand NOS activity during both embryonic development and metamorphosis.

In this paper, we determined *Ciona* NOS, at the gene and protein levels, and NO localization profiles during larval development. We then compared them with the activity of *Nos* genomic regulatory sequences that drive its expression in specific territories of the larva. Overall, this study provides novel insights for NOS regulation in *Ciona* allowing a more detailed comparative analysis of the evolution of this pathway from invertebrates to vertebrates.

## 2. Results

Despite several studies on the involvement of NO in ascidian metamorphosis, its role in the different stages of larval development and in the life-cycle transition from larva to adult is still poorly defined [34,36,40,43,45]. We performed comparative analyses of the spatial expression profiles of NOS and NO production along the larval body axis in *C. robusta* to fully define the territories of *Nos* gene and protein expression, and NO diffusion. This analysis is instrumental to further understand the function of NO in ascidian larvae and to perform an exhaustive evolutionary comparison of its role during metamorphosis.

By using in situ hybridization, immunochemistry, and biochemical methods to detect NOS expression and NO messenger diffusion territories, we have taken a snapshot of the *Ciona* larval NO pathway localization. In addition, three *No*s genomic fragments previously identified by Caccavale et al. [32] were used to fully define the NOS localization pattern.

### 2.1. NOS and NO in the Larval Palps

**Figure 1:**
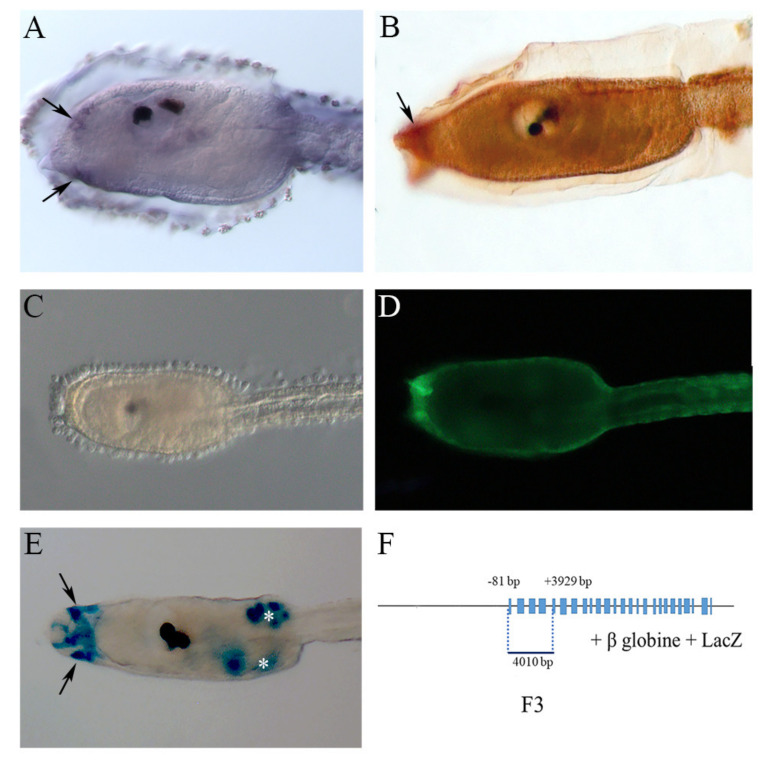
NOS and NO localization in *Ciona* larval palps (arrows). (**A**) In situ hybridization of *Nos* gene in sensory vesicle at the early to mid-larval stage. (**B**) Immunochemistry assay of NOS, using antibody uNOS, at the late larval stage. (**C**,**D**) NO localization using DAF-FM-DA at the late larval stage. Bright field (**C**) and dark field (**D**) of the same larva. (**E**) Transgenic assay of *Ciona* F3 *Nos* regulatory region. (**F**) Schematic representation of F3 construct and F3 regulatory region position along the *Nos* gene locus. The white asterisk indicates an ectopic signal in mesenchymal cells.

*Nos* expression starts at the early to mid-larval stage in the most anterior part of the embryo, in the ventral and dorsal epidermis of the palps (Figure 1A). At the next stage of the late larva, we observed localization of the NOS enzyme in the palps by diaminobenzidine immunochemistry (Figure 1B) whereas, at this stage, the transcript rapidly disappears from the palps, thus indicating the dynamic character of *Ciona* NOS expression during development. From the mid- to late-larval stages, the presence of the gaseous NO fluorescent signal, revealed by using the NO-specific indicator DAF-FM-DA, remains localized in the palps (Figure 1D). NOS localization in these sensory structures was further confirmed by the activity of the F3 *Nos* regulatory region (Figure 1E,F). This genomic fragment extending from the fourth exon to position −80 upstream of the translation start codon (Figure 1F) directs LacZ reporter gene expression in the epidermis of the palps.

The late larval stage represents a pre-metamorphic developmental phase and palps constitute a transient sensory adhesive organ that assures larval settlement and the onset of metamorphosis. NOS and NO expression in structures devoted to substrate attachment indicates the possible role of this signaling pathway for the production of the preliminary signals necessary for settlement.

### 2.2. NOS and NO in the Anterior Nervous System

**Figure 2:**
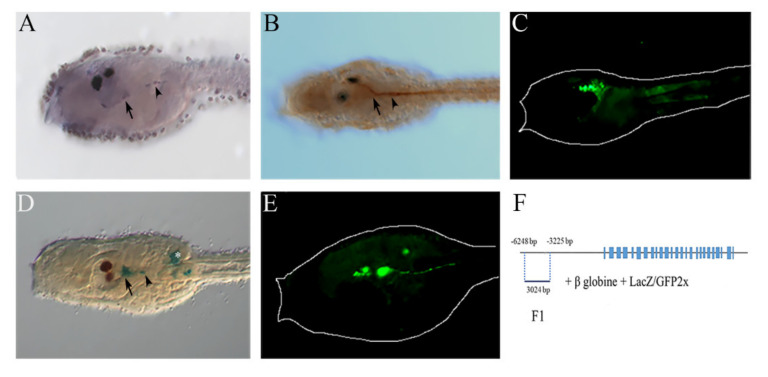
NOS and NO localization in *Ciona* larvae in anterior CNS. (**A**) *Nos* gene expression in posterior sensory vesicle (arrow) and neurons of the motor ganglion (arrowhead) at the early to mid-larval stage. (**B**) Immunochemistry assay of NOS, using antibody uNOS, at the mid-larval stage, showing enzyme expression in the posterior sensory vesicle (arrow) and motor ganglion neurons (arrowhead). (**C**) NO localization using DAF-FM-DA at the mid-larval stage. (**D**,**E**) LacZ and GFP staining of *Ciona* F1 *Nos* regulatory activity in the same regions of the CNS labeled by NOS transcript and enzyme (corresponding arrows and arrowheads). (**F**) Schematic representation of F1 construct and position of F1 regulatory region along the *Nos* gene locus.

Looking at the *Ciona* NOS sequence, its neuronal-type features are evident for the presence of the neuronal PDZ domain typical of the vertebrate NOS1 and of the amphioxus NOSC neuronal enzymes [31,36]. According to its neuronal features, NOS expression at the transcript and protein levels starts, at the mid-larval stage, in the posterior sensory vesicle and in the nerve fibers extending toward the motor ganglion (also called visceral ganglion) (Figure 2A,B). NOS expression in the anterior central nervous system (CNS) is accompanied by NO signal localization in these territories (Figure 2C). *Ciona* F1 *Nos* enhancer shows a similar activity. This regulatory element of about 3 kb, located upstream of the *Nos* gene from position −3225 to −6248, drives reporter gene expression in the posterior region of the cerebral vesicle and in the neural fibers extending toward the motor ganglion (Figure 2D,E). Thanks to the synaptic connectome of the *Ciona* larva, it is evident that the posterior vesicle receives fiber projections from the adhesive organ and epidermal sensory neurons [60,61]. Accordingly, we observed NOS and NO expression in these interneurons of the posterior brain vesicle that extend axons to the motor ganglion where they synapse neurons of the motor system connected to muscles [62,63]. This motor ganglion is considered as a central pattern generator (CPG) of the CNS that integrates sensory inputs for correct modulation of motor outputs [60,64,65].

### 2.3. NOS and NO in the Tail

As larval development proceeds, at the stage of late larva, NOS starts to be localized more posteriorly along the tail. Comparative analyses of NOS/NO territories of expression allowed us to fill the gaps of knowledge about their localization profile along the tail.

**Figure 3:**
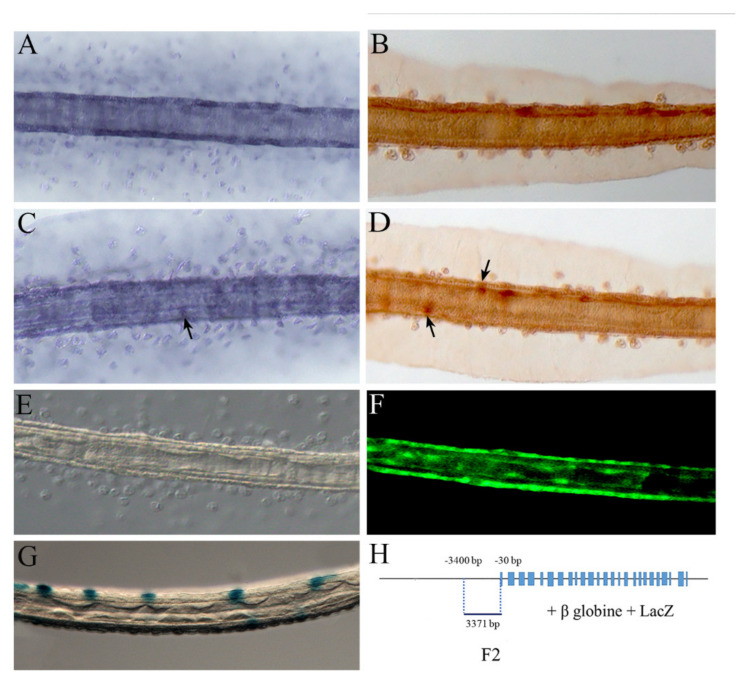
NOS and NO localization in the *Ciona* larval tail. (**A**) *Nos* gene expression in the epidermal cells of the tail at the larval stage by in situ hybridization. (**B**) Immunochemistry assay of NOS, using antibody uNOS, at the late larval stage. (**C**,**D**) NOS gene and protein expression in tail epidermal neurons (arrows) in the larval stage by in situ hybridization and immunochemistry assay, respectively. (**E**,**F**) NO localization by DAF-FM-DA in the epidermis and tail epidermal neurons of a late larval tail. Bright field (**E**) and dark field (**F**) of the same larva. (**G**) Transgenic assay of *Ciona* F2 *Nos* regulatory region. (**H**) Schematic representation of F2 sequence position along the *Nos* gene locus.

We clearly reveal a novel signal in the sensory epidermal neurons that extend rostro-caudally along the dorsal and ventral axes of the larval tail (Figure 3C–F) and have axons running beneath the epidermis [66]. NOS localization in the sensory epidermal neurons was also obtained by LacZ reporter staining under the control of the F2 *Nos* regulatory region (Figure 3G,H) extending from position −30 to −3400 upstream of the translation start codon.

It is worth noting that the sensory epidermal neurons are part of the peripheral nervous system (PNS) and are fundamental elements of the CPG. In *Ciona* just before settlement, there is evidence for mechanosensory and chemotactic activities of swimming larvae mediated by epidermal sensory neurons. This activity is necessary for the control of neuronal signals and the correct progress of metamorphosis [66,67].

As previously reported in Comes et al. [36], we also observed NOS expression in the epidermal cells (Figure 3A,B), namely in the tail cells that coordinate the coiling of internal tissues during metamorphosis [68,69]. This is again a territory of expression necessary for the correct progression of metamorphosis. Therefore, NOS expression in the tail epidermis remains in the following step of metamorphosis with a precise and dynamic pattern that accompanies progression of tail resorption [36].

## 3. Discussion

### 3.1. NO Signaling in the Different Phases of Metamorphosis

**Figure 4:**
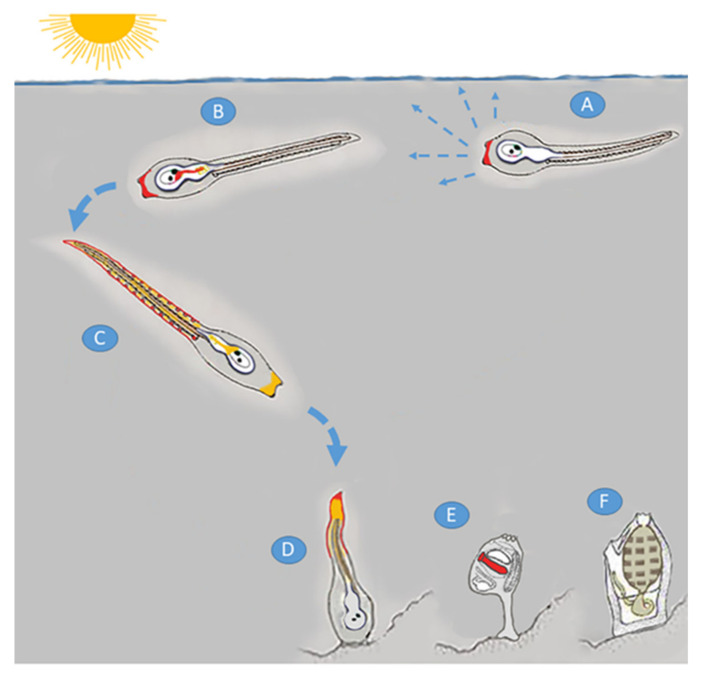
Schematic representation of *Ciona* larval development and metamorphosis. Just after hatching (**A**), the larva swims randomly in all directions. *Nos* transcript localization is visible in the palps (shown in red). At mid-larval stage (**B**), NOS transcript and/or protein expression continue to be visible in the palps and a novel signal appears in the CNS (red color). From the mid- to late-larval stage, *Ciona* becomes photosensitive, changes its swimming behavior, and heads towards the bottom. At the late larval stage (**C**) NOS transcript and protein appear in the epidermis and tail sensory neurons of the PNS. At this stage, only NO is still visible in the palps and CNS (yellow color) and diffuses in mesoderm and notochord. After settlement (**D**), when tail resorption starts, NOS continues to be expressed in the epidermis and NO diffuses not only in the mesoderm and notochord but also concentrates in the reabsorbing tail tip. *Nos* is visible in the newly formed digestive organ at the juvenile stage (red color) (**E**). Now there is an extensive tissue remodeling, and the replacement of most of the larval tissues with the newly forming organs of the adult (**F**).

Our results on NO diffusion and NOS expression, at the transcript and protein level, indicate a very dynamic pattern that follows the different phases of larval development and metamorphosis, from the induction of settlement and control of swimming behavior to tail resorption (Figure 4).

The ascidian larva has a characteristic pattern of swimming. After hatching, the early larvae do not show any photoresponse, swimming upwardly and randomly both in the dark and in the light (Figure 4A) [70]. Our results indicate the presence of NOS and NO in the palps of hatching larvae, thus preparing the larva to the induction of the settlement phase. Subsequently, from the mid- to late-larval stage, the presence of NOS (before) and NO (immediately after) in the posterior sensory vesicle and in the nerve fibers extending toward the motor ganglion indicates the putative role played by NO as a neurotransmitter of the CPG to induce the change in larva swimming behavior (Figure 4B,C). This change, together with the activation of a negative photoresponse, leads larvae to swim downwardly, away from the light (Figure 4C). At this stage, the NO signal continues to be present in the palps that now exert a fundamental sensory role to search for a suitable substrate. Moreover, NOS presence in epidermal sensory neurons could be a further contribution to changing the larval rhythmic motor activity and downward swimming before the beginning of metamorphosis (Figure 4C). NO at this stage was also reported in muscle and notochord cells, probably due to the free diffusion of the gas from the production site of the epidermis to the surrounding cellular districts [36]. This result indicates the wide diffusion of NO along the tail just before the beginning of tail resorption. After larval adhesion to the substrate, tail resorption starts, and a characteristic localization of NOS and NO was observed by Comes et al. [36] in the epidermis and in the tail tip during its resorption (Figure 4D). During this stage of metamorphosis, some larval tissues are destroyed and replaced by other tissues and organs in the juvenile where NOS/NO localization is determined in the newly forming digestive organ (Figure 4E) [36].

Different NO molecular chaperone or downstream targets have been so far identified in different organisms. The NO signaling through the activation of downstream soluble guanylyl cyclase with consequent cGMP production was reported to be operative alone or in association with the heat shock protein 90 (HSP90) during settlement and metamorphosis in several invertebrates [34,35,36,37,38,41,43,47,48,71]. HSP90 is one of the upstream regulators for NOS, it acts as a molecular chaperone and its binding to NOS activates the enzyme and consequent NO production [72,73,74].

In ascidian *C. robusta,* larval development and metamorphosis require a complex interplay of events including, in addition to NO production, the activation of the MAP kinases, JNK and ERK, and apoptosis. An apoptotic wave, originating at the tail extremity, propagates up to the tail base, promoting caspase-3-dependent apoptosis of tunic, epidermis, muscle, and notochord cells [75]. Interestingly, the tail epidermis, where both NOS and NO are present, exerts a prioritized role in the coordination of the apoptotic events which will lead to the death of almost all the cells of the tail epidermis, the muscles, and the posterior notochord [69]. Biochemical experiments have revealed that NO itself is also able to modulate caspase-3-like activity [36], thus driving the apoptotic processes. Regarding the MAP kinases, whereas phosphorylated JNK is present in the CNS of larvae, activated ERK is detected first in the papillae of swimming larvae and later in tail cells before the wave of apoptosis occurs [76]. Interestingly, it has been demonstrated that the activation of both MAP kinases is necessary for the onset of apoptosis and metamorphosis. In addition, NO can positively affect ERK signaling, inducing the down-regulation of the genes encoding the phosphatases mkp1 and mkp3 that are responsible for maintaining the ERK phosphorylation levels necessary for the transcription of downstream genes involved in metamorphosis [45]. The crosstalk of NO with a MAP kinase was also reported in larval settlement of the bryozoan *Bugula neritina* [77]. In *C. robusta*, NO can act on ERK signaling, also modifying the protein by nitration of tyrosine residues. Indeed, nitrated ERK and phospho-ERK were identified during larval development [40]. The occurrence of these nitrated species would suggest protein nitration as a signaling mechanism mediated by oxidative stress-like conditions [40]. Moreover, nitration could also indirectly affect ERK activation, considering that ERK nitration may positively or negatively affect ERK signaling by regulating phosphorylation of specific amino acid residues [40].

### 3.2. Conservation of NOS/NO Signaling during Evolution

The palps are the first larval structure labeled by *Nos*, and the beginning of metamorphosis is induced through the adhesion of the palps to the substrate. This function is fundamental not only in *Ciona* but also in amphioxus, the other non-vertebrate chordate species where NO was reported as a potent morphogen. Its inhibition in amphioxus embryos prevents the opening of the mouth and the correct development of two pharyngeal structures, the endostyle and the club-shaped gland involved in rostral metamorphosis [78]. This function was evidenced also in other marine invertebrates. Indeed, in molluscan larvae NOS is expressed in the anterior foot of *Haliotis asinina* and is fundamental for induction of metamorphosis [79]. Moreover, NOS was detected in the apical ganglia of the snails *Crepidula fornicata* and *Ilyanassa obsoleta,* especially in larvae, before becoming competent to metamorphose [37,80]. These apical structures have been implicated in settlement and metamorphosis regulation in various taxa, and their photo ablation in the nudibranch *Phestilla sibogae* abolishes responses to a natural inducer of metamorphosis [81].

The palps represent not only a primary element for induction of metamorphosis but also the most anterior sensory structure of the embryo. In accordance with a conserved evolutionary role, NO is crucial for the definition of anterior structures in many vertebrate species. The loss of function of *Nos1* arrests mouth-opening in vertebrate *Xenopus* and zebrafish and induces severe defects in pharyngeal arch patterning and aberrant cartilages and bones formation [82].

In the following phase of larval development, *Nos* expression starts in specific regions of the CNS corresponding to the anterior neuronal complex formed by motor neurons with a cholinergic phenotype and GABA/glycinergic interneurons, connected to caudal sensory neurons. These neural domains are compatible with a simple CPG producing a rhythmic movement of the tail [63] and may represent the ancestral state of the vertebrate motor system. Therefore, expression in the relay neurons of the motor ganglion at the mid-to late larval stage could be indicative of a role of NO in regulating the swimming program that precedes the induction of metamorphosis. At this stage, a shadow response gives rise to the larva swimming downwardly to find appropriate substrates for settlement [70]. This feature starts developing at 1.5 h after hatching, and requires an increase in tail-beat frequency [83] to give rise to bilateral contractions of the muscle bands on either side of the notochord and to generate helical swimming with symmetrical undulations of the tail. Interestingly, a similar role for NO in the control of swimming behavior has been evidenced also in other organisms from gastropods to vertebrate species. In the cnidarian *Aglantha digitale* and in the gastropod *Melibe leonina*, the NO signaling pathway, acting via a cGMP-dependent mechanism, modulates the rhythmic swimming associated with feeding [25,84]. This behavior is conserved during evolution up to vertebrates. In lamprey, NO plays an important role in motor control, increasing locomotor burst frequency via modulation of both excitatory and inhibitory transmission [85]. Moreover, the swimming CPG in the spinal cord of *Xenopus* tadpoles is modulated by NO which affects both the duration and intensity of swimming acting on glycinergic and GABAergic transmission [86,87]. In *Ciona* the activation of this swimming program is strictly connected with the induction of metamorphosis. It is a first fundamental step necessary to approach the substrate and begin settlement.

The correlation between NO and the control of rhythmic motor activity and swimming speed induced by CPG is further supported by the presence of NOS and NO in the tail sensory neurons. A role for NO in the control of mechanosensory processing has been well-described in both invertebrates and vertebrates [85,88,89,90,91]. It was already evidenced in the invertebrate locust, where it was suggested that NO acted as a modulator of the input from the sensory neurons and the output of motor neurons [92,93].

NOS and NO expression in the PNS is a highly conserved characteristic common to other chordate species. The identification of both *Ciona* and amphioxus *Nos* regulatory elements able to recapitulate endogenous expression in tail sensory neurons lead to suggest a high level of conservation of these genes [32]. These common territories of expression in the nervous system between phylogenetically extant species suggest common conserved functions for correct progression of their metamorphosis.

Furthermore, this pattern has been conserved also in vertebrates where NOS1 modulates, in a more articulated pattern, synaptic transmission and neuroplasticity in sensory related areas of the brain and in the enteric PNS [94,95]. In the vertebrate *Xenopus* tadpoles, NO has a role in potentiation of synaptic transmission during the descending activation of the swimming CPG. In particular, in several species of anuran and urodel amphibians NO controls the modulatory pathways to switch from ondulatory to free-swimming locomotion style during metamorphosis [88,96,97,98].

Finally, the epidermal cells also represent an evolutionary conserved territory of NOS/NO expression where this signalling acquired multiple novel functions in vertebrates. Indeed, NO was reported to be highly produced in the epidermis of some amphibians during development [99,100,101]. Its presence in specific epidermal cells in *Xenopus laevis* suggested NO involvement in epidermis development as well as in physiological tadpole ammonium release [4,100]. Recently, NO was shown to be required for epidermal permeability barrier homeostasis in mice [102]. In humans, NO is produced in many cell types of the skin and has important roles in a variety of physiological and pathological processes [103]. In particular, the keratinocytes, the predominant cell type of the epidermis, constitutively express the neuronal isoform of NO synthase, NOS1.

## 4. Conclusions

In conclusion, the picture emerging from these studies indicates the central role played by NO in *Ciona* as a neurotransmitter and regulator of swimming behavior. This role has been highly conserved during evolution and in vertebrates has been also coopted to accomplish the same regulatory functions in a wider variety of physiological processes.

In *Ciona*, NOS/NO signaling is necessary to induce settlement and to trigger metamorphosis. The complex and dynamic NOS/NO profile is not only tightly and precisely controlled in time and space but also intimately connected to obtain the final goal of a successful metamorphosis. In other words, NO appears to be one of the key molecules for the correct fate of the larva, acting on the decision to adhere to a substrate suitable for metamorphosis and operating on the transformation process itself to become an adult organism. RNAi studies will be necessary to highlight the changes when NO is silenced and to identify downstream targets of NO. Overall, this functional approach will allow researchers to dissect and fully determine NOS and NO mechanisms of action in every single step of ascidian metamorphosis.

## 5. Materials and Methods

### 5.1. Animal Care and Ethical Statement

Adult *C. robusta* were collected from the Gulf of Naples and Gulf of Taranto (Italy). They were reared for at least 1 week in an animal facility in 35-L tanks with flow-through seawater. Animals were fed continuously with a mixture of algae. Eggs and sperm from several specimens were gathered in sterile conditions and separately for in vitro cross-fertilization [104,105]. Embryos and larvae were staged following the developmental timeline established by Hotta et al. [106]. All procedures were in compliance with current available regulations for the experimental use of live invertebrate animals.

### 5.2. Genomic Fragments Amplification and Preparation of Enhancer DNA Constructs

Three partially overlapping genomic fragments encompassing *Nos* gene locus from the fourth exon to position −6248 bp upstream of the ATG were used in transgenic assays to analyze their enhancer activity. The DNA fragments were amplified by PCR from *C. robusta* genomic DNA and were cloned as reported by Caccavale et al. [32]. The LacZ expression construct was made by using the pBluScript II KS containing the human beta-globin basal promoter upstream to the LacZ reporter gene and the SV40 polyA sequence [107]. Similarly, the GFP expression construct was prepared by using the pSP72 vector (Promega, Madison, Wisconsin, USA) containing the GFP gene and SV40 polyA, as described by Zeller et al. [108], to which we added the human beta-globin basal promoter.

### 5.3. In Situ Hybridization

Whole-mount in situ hybridization on different larval stages was performed as previously described [36].

### 5.4. NO Detection

NO localization was performed by using 4-amino-5-methylamino-2′,7′-difluorofluorescein diacetate (DAF-FM-DA) (Molecular Probes, Eugene, Oregon, USA) which becomes fluorescent when it reacts with NO-derived species [36].

### 5.5. Immunochemistry

Larvae were fixed in cold methanol for 10 min. After two successive washings with cold methanol, larvae were kept at −20 until use. Samples were hydrated by washings with aqueous solutions containing decreasing concentrations of methanol until reaching 25%. Larvae were washed in TBS pH 8.8 + Triton 0.25% (2 × 15 min) and then blocked for 40 min with 50% NGS in TBS pH 8.8 + Triton 0.25% (TBST). Larvae were incubated at 4 °C for 43 h with the primary antibody uNOS (Affinity BioReagents, Invitrogen, Waltham, Massachusetts, USA), 1:100 in 50% NGS-TBST. After washings with TBST, larvae were incubated for 1 h in 1% BSA in TBST and then incubated overnight at 4 °C with anti-rabbit IgG biotinylated secondary antibody, 1:250 in TBST. Incubation with the ABC kit (Vector Laboratories, Burlingame, CA, USA) and the DAB (3,3′-diaminobenzidine) solution was performed according to manufacturer instructions. As control, omission of primary antibody was performed.

## Data Availability

All data is available upon request.
